# Prognostic signature of *protocadherin 10* methylation in curatively resected pathological stage I non-small-cell lung cancer

**DOI:** 10.1002/cam4.507

**Published:** 2015-08-15

**Authors:** Hiroaki Harada, Kazuaki Miyamoto, Yoshinori Yamashita, Kiyomi Taniyama, Kazuko Mihara, Mitsuki Nishimura, Morihito Okada

**Affiliations:** 1 Department of Respiratory Surgery, National Hospital Organization Kure Medical Center/Chugoku Cancer Center3-1 Aoyama-cho, Kure, Hiroshima, 737-0023, Japan; 2 Institute for Clinical Research, National Hospital Organization Kure Medical Center/Chugoku Cancer Center3-1 Aoyama-cho, Kure, Hiroshima, 737-0023, Japan; 3 Department of Surgery, National Hospital Organization Higashihiroshima Medical Center513 Teraya Saijyo-cho, Higashihiroshima, Hiroshima, 739-0041, Japan; 4 Department of Surgical Oncology, Research Institute for Radiation Biology and Medicine, Graduate School of Biomedical Science, Hiroshima University1-2-3 Kasumi Minami-ku, Hiroshima, Hiroshima, 734-8551, Japan

**Keywords:** Methylation, molecular biomarker, non-small-cell lung cancer, prognosis, protocadherin 10, surgery

## Abstract

Although curative resection is the current treatment of choice for localized non-small-cell lung cancer (NSCLC), patients show a wide spectrum of survival even after complete resection of pathological stage I NSCLC. Thus, identifying molecular biomarkers that help to accurately select patients at high risk of relapse is an important key to improving the treatment strategy. The purpose of this study was to evaluate the prognostic signature of *protocadherin 10* (*PCDH10*) promoter methylation in curatively resected pathological stage I NSCLC. Using methylation-specific polymerase chain reaction assays, methylation of *PCDH10* promoter was assessed in cancer tissues of 109 patients who underwent curative resection of pathological stage I NSCLC. Associations between *PCDH10* methylation status and disease outcome was analyzed. *PCDH10* promoter methylation was detected in 46/109 patients (42.2%). Patients with methylated *PCDH10* showed significantly worse recurrence-free, overall, and disease-specific survival compared with those without methylation (*P* < 0.0001, *P* = 0.0004, *P* = 0.0002, respectively). Multivariate Cox proportional hazard regression analysis revealed that adjusted hazard ratios of methylated *PCDH10* were 5.159 for recurrence-free, 1.817 for overall, and 5.478 for disease-specific survival (*P* = 0.0005, *P* = 0.1475, *P* = 0.0109, respectively). The pattern of recurrence was not significantly different between patients with and without *PCDH10* methylation (*P* = 0.5074). *PCDH10* methylation is a potential biomarker that predicts a poor prognosis after curative resection of pathological stage I NSCLC. Assessment of *PCDH10* methylation status might assist in patient stratification for determining an appropriate adjuvant treatment and follow-up strategy.

## Introduction

Because non-small-cell lung cancer (NSCLC) is a molecularly heterogeneous disease, even patients with the same tumor, node, metastasis (TNM) classification in the American Joint Committee on Cancer (AJCC)/Union for International Cancer Control (UICC) staging system show varied clinical outcomes and prognoses. Although surgical resection for early stage lung cancer is the best treatment for cure, even after curative resection for pathological stage I NSCLC, ∼30% of patients eventually relapse after surgery 1. For improved prognosis after resection, adjuvant chemotherapy has been widely applied; however, prospective randomized data showed that adjuvant chemotherapy for stage IB does not significantly improve survival; in fact, a detrimental effect was observed for stage IA 2–4. For effective application of adjuvant therapy, identifying a novel biomarker able to predict patients with high chance of relapse is important in establishing a tailored treatment strategy for stage I NSCLC. Recent cumulative evidence indicates that aberrant promoter methylation and subsequent silencing of various specific genes play important roles in the development and/or progression of many human cancers 5–9, and promoter methylation status of specific genes has been reported as a promising biomarker for predicting disease outcome more accurately than TNM staging 10–12.

Protocadherins are a subfamily of the cadherin superfamily of genes that exhibit cell-to-cell adhesion activity with a mechanism thought to be distinct from that of classic cadherins 13,14. Protocadherin 10 (PCDH10) belongs to the non-clustered protocadherin and is reported to be widely expressed but frequently silenced by promoter methylation in many carcinomas and hematological malignancies. Recent literature demonstrated that PCDH10 exerts a tumor-suppressor effect in gastric cancer by inducing apoptosis, controlling cell growth, and inhibiting cell invasion and metastasis 15; however, the prognostic value of epigenetic silencing of PCDH10 in early-stage lung cancer remain to be investigated.

In this study, we employed a methylation-specific polymerase chain reaction (MS-PCR) assay to examine *PCDH10* methylation status in curatively resected pathological stage I NSCLC. To evaluate the prognostic signature of *PCDH10* promoter methylation in patients with curatively resected pathological stage I NSCLC, we analyzed whether methylation status is related to postoperative prognosis. We also assessed the association between *PCDH10* methylation and clinicopathological features to investigate whether *PCDH10* methylation is an independent prognostic marker.

## Patients and Methods

### Study population

This study was approved by the Institutional Review Boards of Kure Medical Center/Chugoku Cancer Center. In total, 109 patients who underwent complete resection for pathological stage I NSCLC at Kure Medical Center/Chugoku Cancer Center between June 2005 and November 2011 were enrolled. Detailed enrollment criteria were described previously 16. Briefly, patients who received adjuvant therapy were included, and exclusion criteria were: neoadjuvant therapy, Noguchi Type A or B tumors (these types are not considered as candidates for adjuvant therapy because of their extremely excellent prognoses), small tumors precluding further sample extraction subsequent to routine histological examination, lost to follow-up or death from non cancerous causes <100 days postoperatively.

Video-assisted thoracic surgeries were performed in all patients. Systemic or selective lymph node dissection was conducted in patients who underwent lobectomy or segmentectomy. Wedge resection without lymph node dissection or concomitant with lymph node sampling was performed in selected patients, primarily because they had limited lung/cardiac function or multiple comorbidities.

The outpatient follow-up protocol was as described previously 16. Briefly, chest and upper abdominal computed tomography were performed at 6-month intervals during the first 2 postoperative years and every year thereafter for at least 5 years. Other imaging examinations were performed as needed when patients presented with specific symptoms, or periodically at the discretion of the attending physician. For pulmonary nodules discovered during follow-up, diagnoses were determined by the institutional cancer board as previously described 16, primarily based on the modified Martini and Melamed classification scheme 17,18. For metachronous adenocarcinomas with the same grade of differentiation as the primary tumor, secondary tumors lacking bronchoalveolar carcinoma components were classified as pulmonary metastases. Recurrence patterns were defined as previously described 16; local recurrence was defined as the presence of a recurrent tumor in the same lobe or lymph node in the same ipsilateral hemithorax as the primary tumor. Pulmonary metastasis in a different lobe, even in the same ipsilateral hemithorax as the primary tumor, was defined as remote recurrence.

### Sample preparation, bisulfite modification, MS-PCR, and immunohistochemical analysis

Sample preparation, bisulfite modification, and MS-PCR were conducted as previously described 12,16,19–22. Briefly, for sodium bisulfite modification, DNA was digested, using *Bam*HI (New England Biolabs, Beverly, MA), and 1 *μ*g of the digested DNA was denatured in 0.3 N NaOH at 37°C for 15 min. The samples underwent 15 cycles of 30-sec denaturation at 95°C and 15-min incubation at 50°C in 3.1 N sodium bisulfite (pH 5.0) and 0.5 mmol/L hydroquinone. The product was desalted with the Wizard DNA cleanup system (Promega, Madison, WI) and desulfonated in 0.6 N NaOH. MS-PCR was carried out using 1 *μ*L of the sodium bisulfite-modified DNA. The primer sequences used for *PCDH10* were: unmethylated forward primer (5′-AGAGTTTTGTTTTGTTTTGTTT-3), unmethylated reverse primer (5′-CACCCACCAAACTACCA-3), methylated forward primer (5′-AGTTTTGTTTCGTTTCGTTC-3), and methylated reverse primer (5′-CCCACCGAACTACCG-3). PCR was performed using 37 cycles with an annealing temperature of 60°C. All procedures were repeated at least four times for each sample. As controls, SssI methylase-treated (New England Biolabs) and methylase-untreated DNA samples from healthy individuals were used. Immunohistochemical staining was conducted as previously described 12,16. A monoclonal antibody (1:1000; Santa Cruz Biotechnology, Santa Cruz, CA) was used to detect PCDH10 protein for immunohistochemical analysis.

### Statistical analysis

Comparisons were performed using the JMP for Windows statistical software (version 9.02; SAS Institute, Cary, NC). Associations between clinicopathological characteristics and *PCDH10* methylation were determined, using Fisher exact test. Using the Kaplan–Meier method, the effect of *PCDH10* methylation status on the time to death or recurrence was estimated, and differences between groups were analyzed by log-rank testing. Recurrence-free survival (RFS), overall survival (OS), and disease-specific survival (DSS) times were defined from the date of surgery until the date of event or the last follow-up. Cox proportional regression analysis was used to analyze the hazard ratios (HRs) of independent factors for survival. Adjusted HRs were calculated using factors that were significant by univariate analysis. Differences were considered significant for *P* < 0.05.

## Results

### *PCDH10* methylation status in the evaluation set

As the evaluation set, *PCDH10* methylation status was analyzed in selected patients, who were matched with respect to gender, age, histology, stage, and differentiation. In this setting, patients with recurrence showed a significantly higher frequency of *PCDH10* methylation compared to those without recurrence (data not shown).

### Correlation between *PCDH10* methylation and clinicopathological features

**Figure 1:**
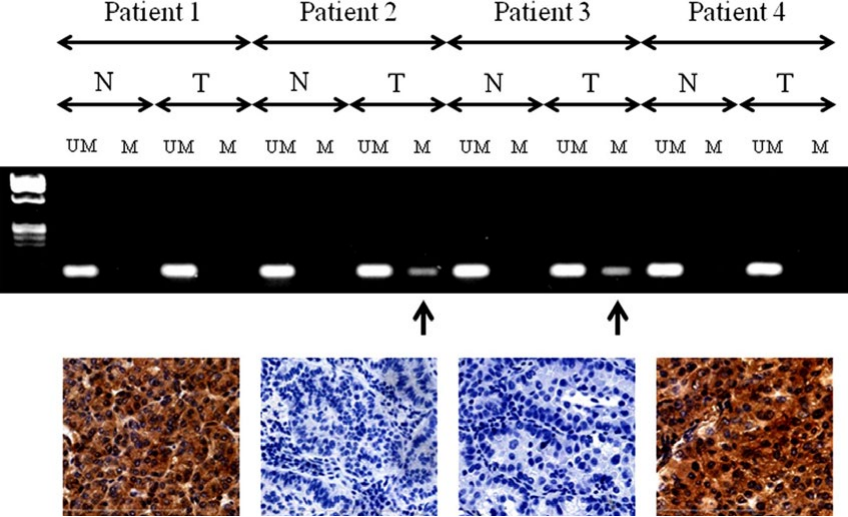
Methylation-specific PCR and immunohistochemical staining for PCDH10. *PCDH10* methylation is detected in tumor tissue from patients 2 and 3 (arrows). Representative expression or lack of expression of PCDH10 according to *PCDH10* methylation status is shown for each of the patients. Original magnification 400×. M, methylated *PCDH10*; N, adjacent normal lung tissue; T, tumor tissue; U, unmethylated *PCDH10*; PCR, polymerase chain reaction; *PCDH10*, *protocadherin 10*.

Representative results of MS-PCR are shown in Figure1. The overall frequency of *PCDH10* promoter methylation was 42.2% (46/109). The demographic and clinicopathological data of patients according to *PCDH10* methylation are shown in Table1.

**Table 1:** Demographic and clinical characteristics of enrolled patients according to *PCDH10* methylation status

	*PCDH10*		*P* value
	Methylation (+)*n* = 46	Methylation (−)*n* = 63	
Age (years)			
Mean ± SD	73.1 ± 8.0	68.9 ± 9.8	0.0199
>70	29	30	
≤70	17	33	0.1236
Sex			
Female	12	30	
Male	34	33	0.0287
Histology			
AD	28	50	
SQ	15	8	
Others	3	5	0.0429
Differentiation			
Well	16	36	
Moderate	22	16	
Poor	8	11	0.0365
Lymphovascular infiltration			
+	18	17	
−	28	46	0.2150
Size			
Mean ± SD	2.85 ± 1.2	2.35 ± 1.1	0.0260
≤3 cm	31	51	
>3 cm	15	12	0.1204
Pleural invasion			
+	10	7	
−	36	56	0.1816
Pathological stage			
IA	24	45	
IB	22	18	0.0461
Smoking			
Current, former	36	38	
Never	10	25	0.0619

*PCDH10*, *protocadherin 10*; AD, adenocarcinoma; SQ, squamous cell carcinoma.

**Figure 2:**
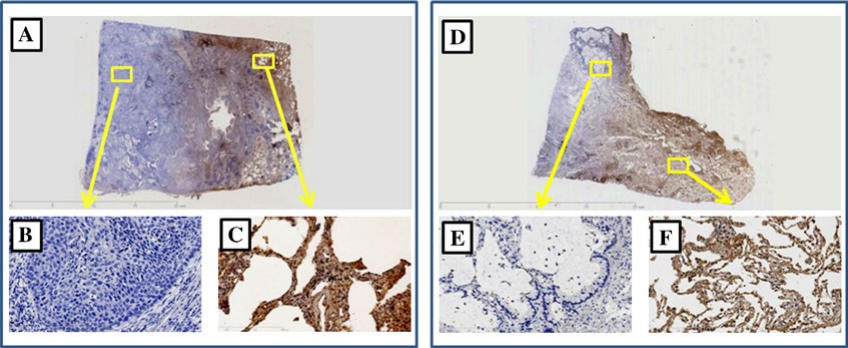
Immunohistochemical staining for protocadherin 10 (PCDH10) in resected specimens (A and D). Tumor tissues with PCDH10 methylation show a lack of PCDH10 immunoreactivity (B and E), and normal adjacent lung tissues with PCDH10 expression are shown as the positive control (C and F).

Although we focused on methylation of the *PCDH10* gene as a biomarker, we also performed immunohistochemical staining of the PCDH10 protein in several patients; representative results of this analysis are shown in Figures1 and 2, which demonstrate that tumors with *PCDH10* methylation showed a marked reduction in PCDH10 protein expression compared to those without methylation or noncancerous tissue.

### Association of *PCDH10* methylation status and disease outcome

**Figure 3:**
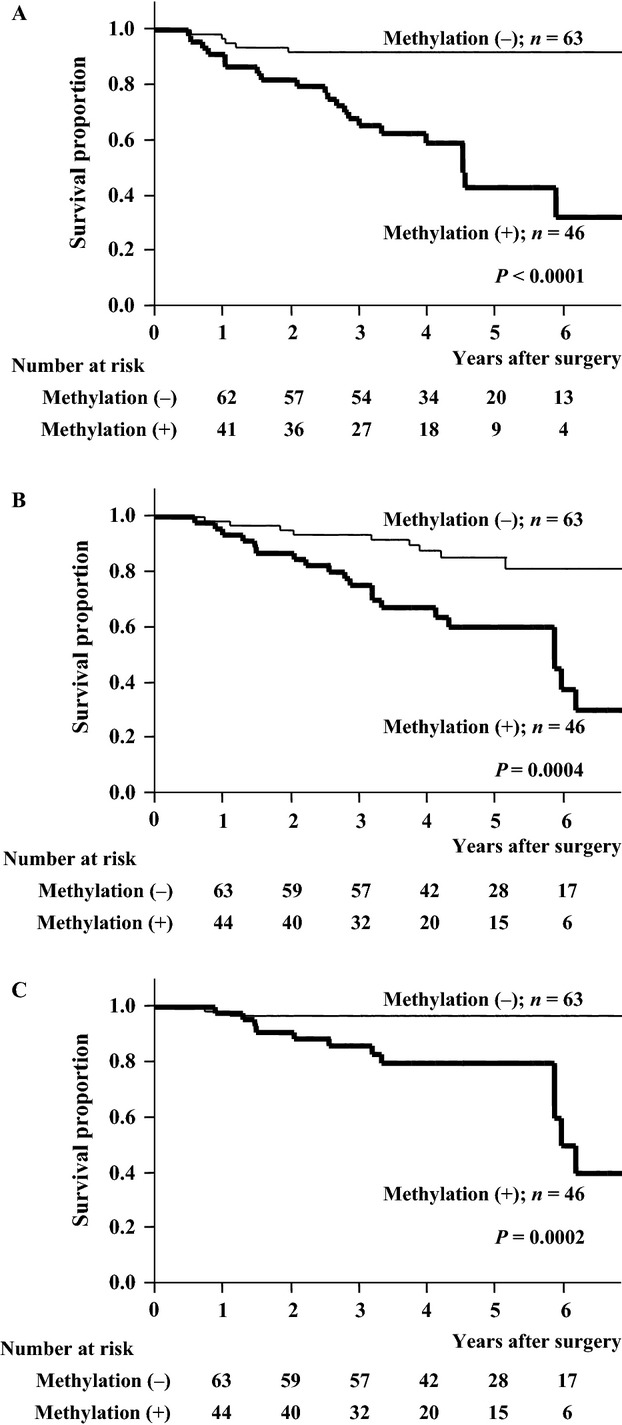
Recurrence-free (A), overall (B), and disease-specific (C) survival curves of patients according to *protocadherin 10* (*PCDH10*) methylation status. Significant differences were detected between patients with and without *PCDH10* methylation (*P* < 0.0001, *P* = 0.0004, and *P* = 0.0002 in A–C, respectively).

The mean follow-up time of all patients was 1584 ± 681 days and that of nondeceased patients was 1751 ± 610 days. Five-year RFS, OS, and DSS rates for all patients were 71.1%, 74.8%, and 90.5%, respectively. The 5-year RFS rate was 43.0% in patients with *PCDH10* methylation, which was significantly worse than 92.1% in patients without methylation (*P* < 0.0001, Fig.3A). Table2 shows the crude and adjusted HRs for RFS. Multivariate analysis revealed that *PCDH10* methylation (*P* = 0.0005), age (*P* = 0.0143), and adjuvant therapy (*P* = 0.0071) were significantly independent poor prognostic factors for RFS. Table3 shows that the recurrence pattern was not significantly different between patients with and without *PCDH10* methylation (*P* = 0.5074). The 5-year OS rate was 60.1% in patients with *PCDH10* methylation, which was significantly worse than 85.3% in patients without methylation (*P* = 0.0004, Fig.3B). As shown in Table4, multivariate analysis revealed that age (*P* = 0.0004) and histology (*P* = 0.0031) were significant independent risk factors; however, *PCDH10* methylation was not an independent prognostic factor (*P* = 0.1475). The 5-year DSS rate was 79.7% in patients with *PCDH10* methylation, which was significantly worse than 96.8% in patients without methylation (*P* = 0.0002, Fig.3C). As shown in Table5, multivariate analysis showed that *PCDH10* methylation (*P* = 0.0109), age (*P* = 0.0452), and histology (*P* = 0.0386) were significantly independent risk factors for DSS.

**Table 2:** Crude and adjusted HR for recurrence-free survival of patients with stage I non-small-cell lung cancer

	Crude HR	*P* value	Adjusted HR	*P* value
Age (years)				
>70	1.000 (reference)		1.000 (reference)	
≤70	0.253 (0.092–0.592)	0.0011	0.311 (0.107–0.799)	0.0143
Sex				
Female	1.000 (reference)			
Male	1.096 (0.507–2.499)	0.8190		
Histology				
AD	1.000 (reference)		1.000 (reference)	
SQ, others	2.417 (1.073–5.287)	0.0337	2.100 (0.897–4.832)	0.0860
Differentiation				
Well, moderate	1.000 (reference)			
Poor	1.761 (0.689–3.995)	0.2212		
Lymphovascular infiltration				
+	1.000 (reference)			
−	0.687 (0.319–1.514)	0.3439		
Size				
<3 cm	1.000 (reference)			
>3 cm	1.288 (0.530–2.848)	0.5562		
Pleural invasion				
+	1.000 (reference)		1.000 (reference)	
−	0.375 (0.168–0.916)	0.0326	0.686 (0.291–1.750)	0.4142
Adjuvant therapy				
+	1.000 (reference)		1.000 (reference)	
−	4.470 (1.331–27.789)	0.0118	5.387 (1.497–34.736)	0.0071
Operative procedure				
Lobectomy, segmentectomy	1.000 (reference)		1.000 (reference)	
Wedge resection	3.069 (1.353–6.795)	0.0082	1.269 (0.520–3.055)	0.5957
Pathological stage				
IA	1.000 (reference)			
IB	1.870 (0.866–4.009)	0.1092		
Smoking				
+	1.000 (reference)			
−	0.640 (0.251–1.451)	0.2964		
*PCDH10* methylation				
No	1.000 (reference)		1.000 (reference)	
Yes	6.444 (2.732–17.744)	<0.0001	5.159 (1.999–15.433)	0.0005

HR, hazard ratios; *PCDH10*, *protocadherin 10*; AD, adenocarcinoma; SQ, squamous cell carcinoma.

**Table 3:** Distribution of recurrence pattern according to *protocadherin 10* (*PCDH10*) methylation status or operative procedure

	Pattern of recurrence			*P* value
	Local	Local + remote	Remote	
*PCDH10* methylation status				
+	7	3	11	
−	1	2	3	0.5074
Operative procedure				
Lobectomy or segmentectomy	5	2	9	
Wedge resection	3	3	5	0.6220

**Table 4:** Crude and adjusted HR for overall survival of patients with stage I non-small-cell lung cancer

	Crude HR	*P* value	Adjusted HR	*P* value
Age (years)				
>70	1.000 (reference)		1.000 (reference)	
≤70	0.209 (0.077–0.482)	0.0001	0.207 (0.071–0.513)	0.0004
Sex				
Female	1.000 (reference)		1.000 (reference)	
Male	2.873 (1.244–7.804)	0.0120	1.329 (0.446–4.501)	0.6219
Histology				
AD	1.000 (reference)		1.000 (reference)	
SQ, others	4.725 (2.227–10.242)	<0.0001	3.669 (1.542–9.262)	0.0031
Differentiation				
Well, moderate	1.000 (reference)		1.000 (reference)	
Poor	2.677 (1.196–5.624)	0.0182	1.476 (0.577–3.693)	0.4100
Lymphovascular infiltration				
+	1.000 (reference)			
−	0.782 (0.376–1.665)	0.5156		
Size				
<3 cm	1.000 (reference)			
>3 cm	1.983 (0.929–4.083)	0.0755		
Pleural invasion				
+	1.000 (reference)			
−	0.453 (0.208–1.092)	0.0753		
Adjuvant therapy				
+	1.000 (reference)			
−	2.167 (0.841–7.354)	0.1160		
Operative procedure				
Lobectomy, segmentectomy	1.000 (reference)			
Wedge resection	1.586 (0.652–3.504)	0.2920		
Pathological stage				
IA	1.000 (reference)		1.000 (reference)	
IB	2.978 (1.447–6.352)	0.0031	1.004 (0.394–2.527)	0.9926
Smoking				
+	1.000 (reference)		1.000 (reference)	
−	0.189 (0.045–0.539)	0.0008	1.717 (0.402–9.113)	0.4774
*PCDH10* methylation				
No	1.000 (reference)		1.000 (reference)	
Yes	3.696 (1.752–8.329)	0.0005	1.817 (0.814–4.319)	0.1475

HR, hazard ratios; *PCDH10*, *protocadherin 10*; AD, adenocarcinoma; SQ, squamous cell carcinoma.

**Table 5:** Crude and adjusted HR for disease-specific survival of patients with stage I non-small-cell lung cancer

	Crude HR	*P* value	Adjusted HR	*P* value
Age (years)				
>70	1.000 (reference)		1.000 (reference)	
≤70	0.223 (0.051–0.705)	0.0091	0.283 (0.061–0.975)	0.0452
Sex				
Female	1.000 (reference)			
Male	3.098 (0.968–13.778)	0.0573		
Histology				
AD	1.000 (reference)		1.000 (reference)	
SQ, others	5.636 (1.854–18.199)	0.0026	3.322 (1.066–11.158)	0.0386
Differentiation				
Well, moderate	1.000 (reference)			
Poor	2.791 (0.861–7.964)	0.0835		
Lymphovascular infiltration				
+	1.000 (reference)			
−	0.709 (0.248–2.074)	0.5194		
Size				
<3 cm	1.000 (reference)			
>3 cm	1.861 (0.622–5.175)	0.2528		
Pleural invasion				
+	1.000 (reference)			
−	0.326 (0.112–1.064)	0.0621		
Adjuvant therapy				
+	1.000 (reference)		1.000 (reference)	
−	4.470 (1.331–27.789)	0.0118	2.676 (0.672–18.308)	0.1775
Operative procedure				
Lobectomy, segmentectomy	1.000 (reference)		1.000 (reference)	
Wedge resection	3.069 (1.353–6.795)	0.0082	1.128 (0.296–4.165)	0.8561
Pathological stage				
IA	1.000 (reference)			
IB	1.870 (0.866–4.009)	0.1092		
Smoking				
+	1.000 (reference)			
−	0.640 (0.251–1.451)	0.2964		
*PCDH10* methylation				
No	1.000 (reference)		1.000 (reference)	
Yes	8.194 (2.517–37.038)	0.0003	5.478 (1.453–27.856)	0.0109

HR, hazard ratios; *PCDH10*, *protocadherin 10*; AD, adenocarcinoma; SQ, squamous cell carcinoma.

### Subgroup analysis according to operative procedures

**Figure 4:**
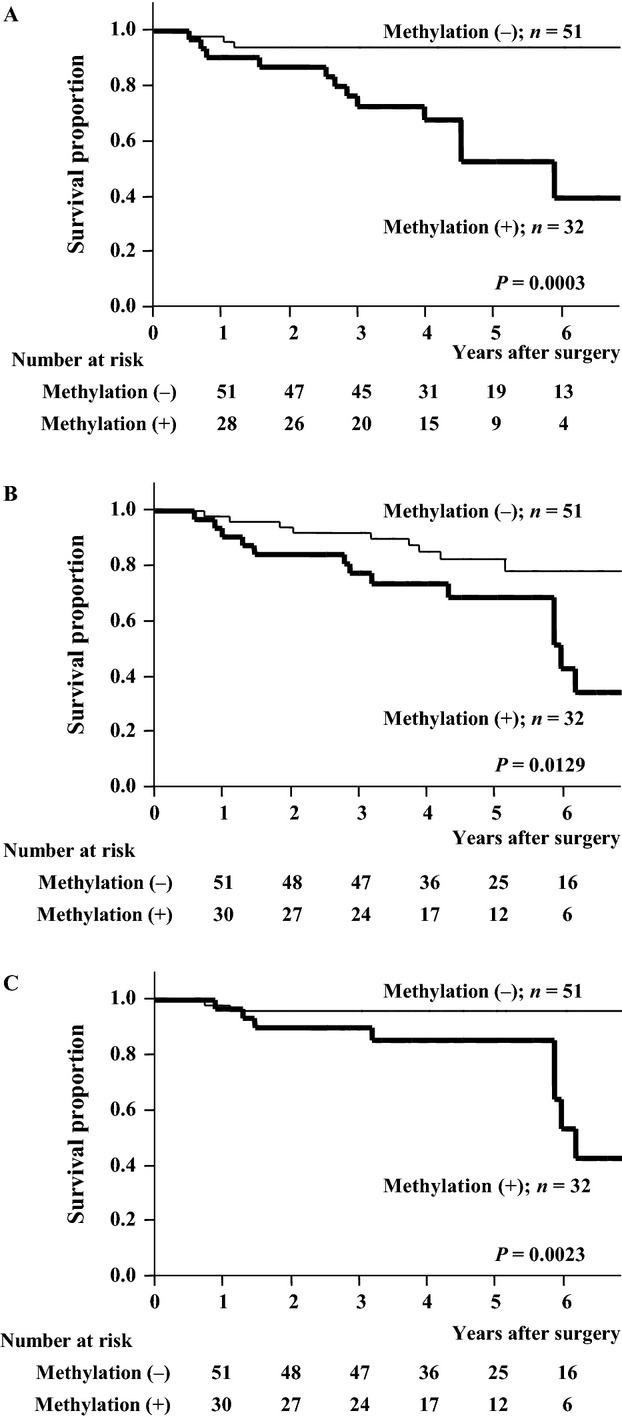
Recurrence-free (A), overall (B), and disease-specific (C) survival curves of patients who underwent lobectomy or segmentectomy. The differences in survival according to *protocadherin 10* methylation status were significant (*P* = 0.0003, *P* = 0.0129, and *P* = 0.0023, respectively).

**Figure 5:**
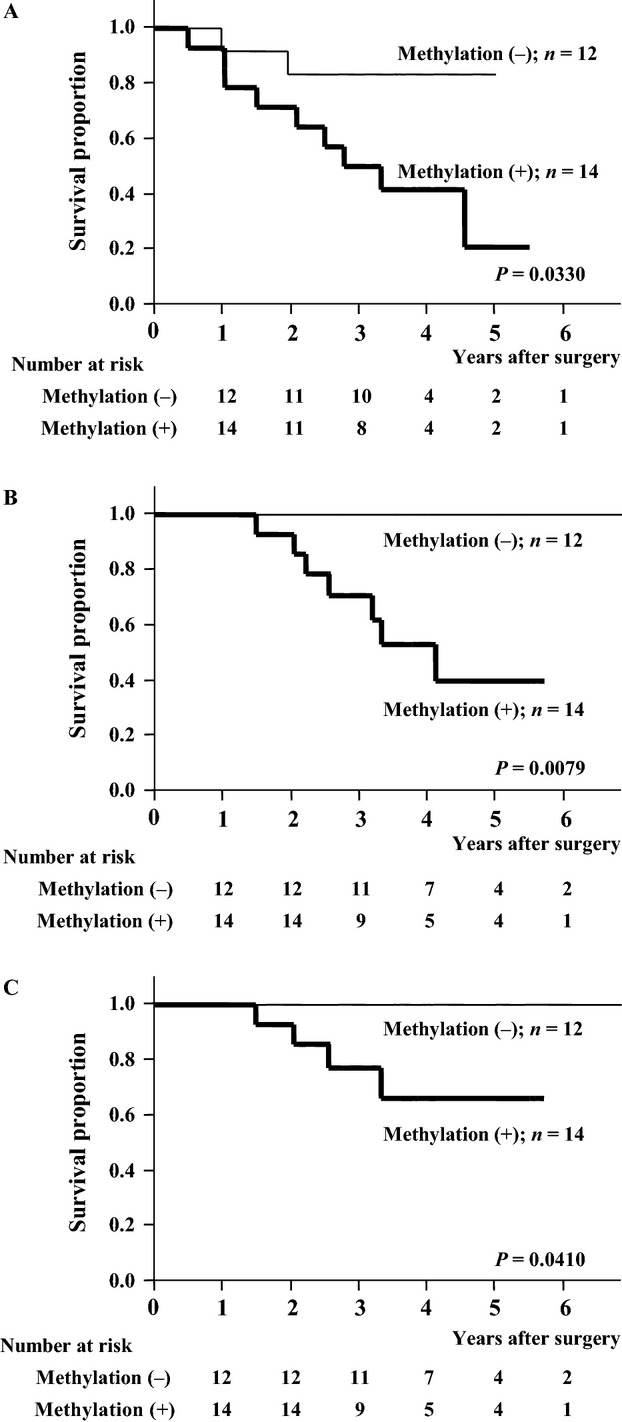
Recurrence-free (A), overall (B), and disease-specific (C) survival curves of patients who underwent wedge resection. The differences in survival according to *protocadherin 10* methylation status were significant (*P* = 0.0330, *P* = 0.0079, and *P* = 0.0410, respectively).

Table3 shows that operative procedure did not significantly impact the recurrence pattern, and Tables2, 4, and 5 show that operative procedure was not an independent prognostic factor for RFS, OS, and DSS in this cohort. However, because systemic lymph node dissection was not performed in patients who underwent wedge resection, a subgroup analysis was performed according to operative procedures. RFS, OS, and DSS were all significantly worse for patients with *PCDH10* methylation, whether the operative procedure was lobectomy/segmentectomy (*P* = 0.0003, *P* = 0.0129, and *P* = 0.0023, respectively; Fig.4A–C) or wedge resection (*P* = 0.0330, *P* = 0.0079, and *P* = 0.0410, respectively; Fig.5A–C).

## Discussion

Cumulative evidence indicates that epigenetic gene silencing of specific genes is associated with the onset and progression of human cancers 8, and the identification of specific gene methylation has been recognized as a powerful molecular biomarker for disease outcome of NSCLC 5,10. Brock et al. showed that the methylation of two or more genes (*p16*, *CDH13*, *RASSF1A*, and *APC*) in tumor tissues and mediastinal lymph nodes of patients with curatively resected stage I NSCLC was associated with a lower 5-year RFS than methylation of fewer than two genes 10. Sandoval et al. demonstrated that hypermethylation of 5 genes (*HIST1H4F, PCDHGB6, NPBWR1, ALX1,* and *HOXA9)* was significantly associated with shorter RFS in these patients 23. The prognostic significance of promoter methylation in several of the above-mentioned genes was also assessed in our cohort (data not shown). Additionally, our group reported that methylation of *BRCA1* and *DLX4* affected disease outcome after curative resection of stage I NSCLC 12,16. Future study by the combined analyses of multiple genes might contribute to identify the gene combination that more accurately predicts disease outcome.

Cadherin-mediated cell–cell interactions are critical events during the development and morphogenesis of cancer 24. Cadherin 1, which is also known as E-cadherin, is widely recognized as a growth and invasion suppressor, and is used even for the diagnosis and prognosis of epithelial cancers. Additionally, the cancer-related functions of several other cadherins have been well studied 25,26. Protocadherin is a member of the cadherin superfamily and contains several domains differing from those of the classical cadherins 25. Although the specific roles of each protocadherin have not been fully investigated, protocadherin genes have been implicated as tumor suppressor genes with important functions, including signal transduction and growth control 27. Several studies have concluded that PCDH10 suppresses tumor cell growth, migration, invasion, and colony formation, and is frequently inactivated epigenetically in colorectal, cervical, nasopharyngeal, esophageal, and other cancer types 27,26,28. Recent study demonstrated an important role of p53 in regulating tumor cell migration through activating PCDH10 expression 29. PCDH10 also appears to induce myeloma cell apoptosis by inhibiting the NF-kappaB pathway 30. Here, although the specific biological role of PCDH10 in NSCLC still needs to be elucidated, we showed that *PCDH10* promoter methylation predicted a poor prognosis in patients with curatively resected pathological stage I NSCLC by performing Kaplan–Meier survival analysis and log-rank testing. To further clarify the prognostic value of *PCDH10* methylation, multivariate analyses were conducted because a significant difference in *PCDH10* methylation status was found in several clinicopathological features in this cohort. Furthermore, a recent study demonstrated that promoter methylation status of another protocadherin (*PCDH20*) was also associated with a shorter OS in lung cancer 31; therefore, the role of other protocadherin methylation in NSCLC needs to be investigated in future studies.

The frequency of *PCDH10* promoter methylation was 42.2% in this study, although Tang et al. reported that promoter methylation of *PCDH10* was observed in 50% of NSCLC tissue and lung cancer cell lines 32. The difference was probably because the present study was limited to patients with pathological stage I NSCLC. In this study, *PCDH10* methylation was found to be an independent prognostic factor for RFS and DSS but not for OS by Cox proportional regression analysis, primarily because the proportion of death that was nondisease related was relatively high in this cohort. Additionally, it is unclear whether adjuvant chemotherapy for patients with *PCDH10* methylation generates a better prognosis, because the number of patients in this study was limited and little is known about the association between PCDH10 and chemoresponse. We demonstrated that marked differences were not found in the patterns of recurrence between patients with and without *PCDH10* methylation in spite of the difference in the risk of recurrence according to *PCDH10* methylation status. It might be inferred from these data that PCDH10 possesses multifunctional tumor-suppressing effects in NSCLC. Although it is known that the prognosis of patients who underwent wedge resection is generally worse than that of patients who underwent anatomical resection (lobotomy or segmentectomy) with lymph node dissection, the subgroup analysis according to the operative procedure revealed that *PCDH10* methylation status was a significant prognostic factor in the both groups of different operative procedures. Future studies using larger sample sizes, including other stages of NSCLC are necessary to more comprehensively elucidate the biological effects of PCDH10 in NSCLC, and to determine whether *PCDH10* methylation status provides clinical information relevant to tailored adjuvant therapy and the postoperative surveillance strategy. Furthermore, since the clinical benefit of DNA demethylating agents is under investigation, clinical trials that assess the benefit of both demethylating agents and standard adjuvant chemotherapy are warranted.

There were several limitations to this study. First, this study was based on the data of patients in a single institution. The number of patients was relatively small, and the mean follow-up time was less than 5 years. Patients who did not undergo systemic lymph node dissection were included, although operative procedure was not an independent prognostic factor and did not significantly impact the recurrence pattern in this cohort. Additionally, the specific mechanism of *PCDH10* methylation for NSCLC progression was not elucidated. Further investigations using larger sample sizes are needed to determine how *PCDH10* methylation contributes to patient outcome.

In summary, this study demonstrated that promoter methylation of *PCDH10* plays a significant role in NSCLC progression and might be a promising prognostic marker for patients with curatively resected pathological stage I NSCLC. Because the significance of postoperative surveillance and adjuvant therapy for patients with curatively resected pathological stage I NSCLC is a matter of great debate, further studies using larger samples are needed to investigate the utility of determining *PCDH10* methylation status of patients with NSCLC.
